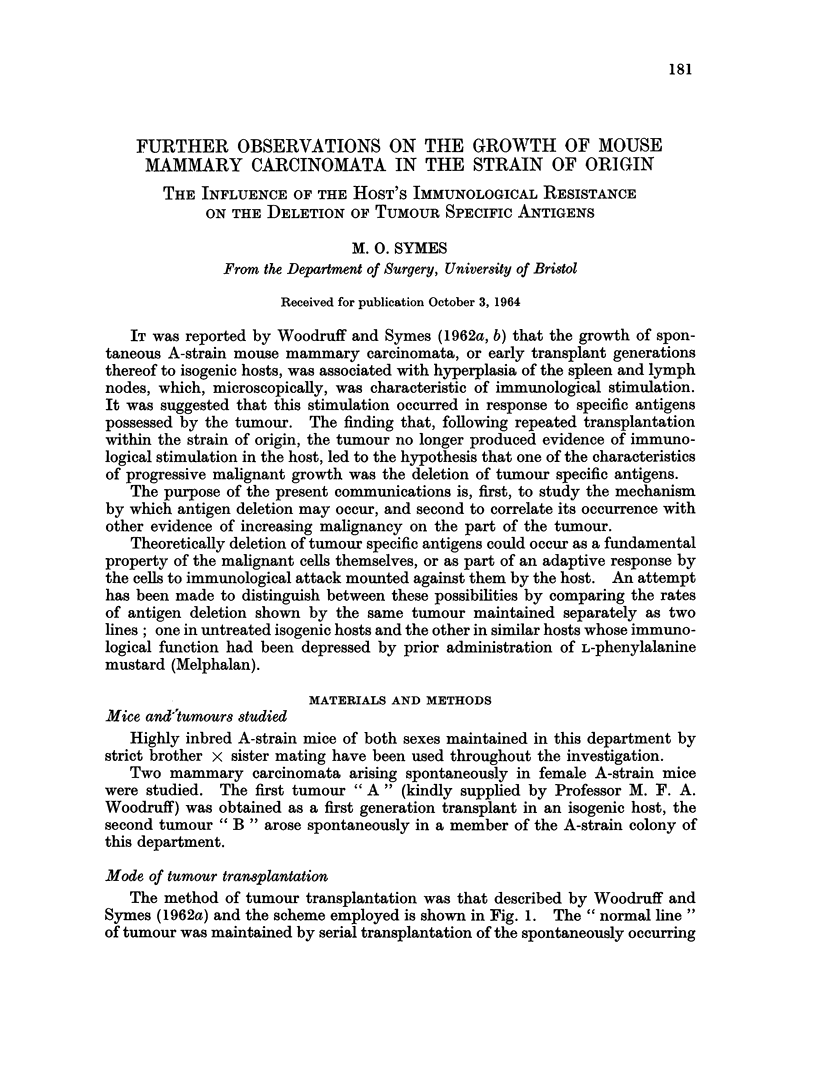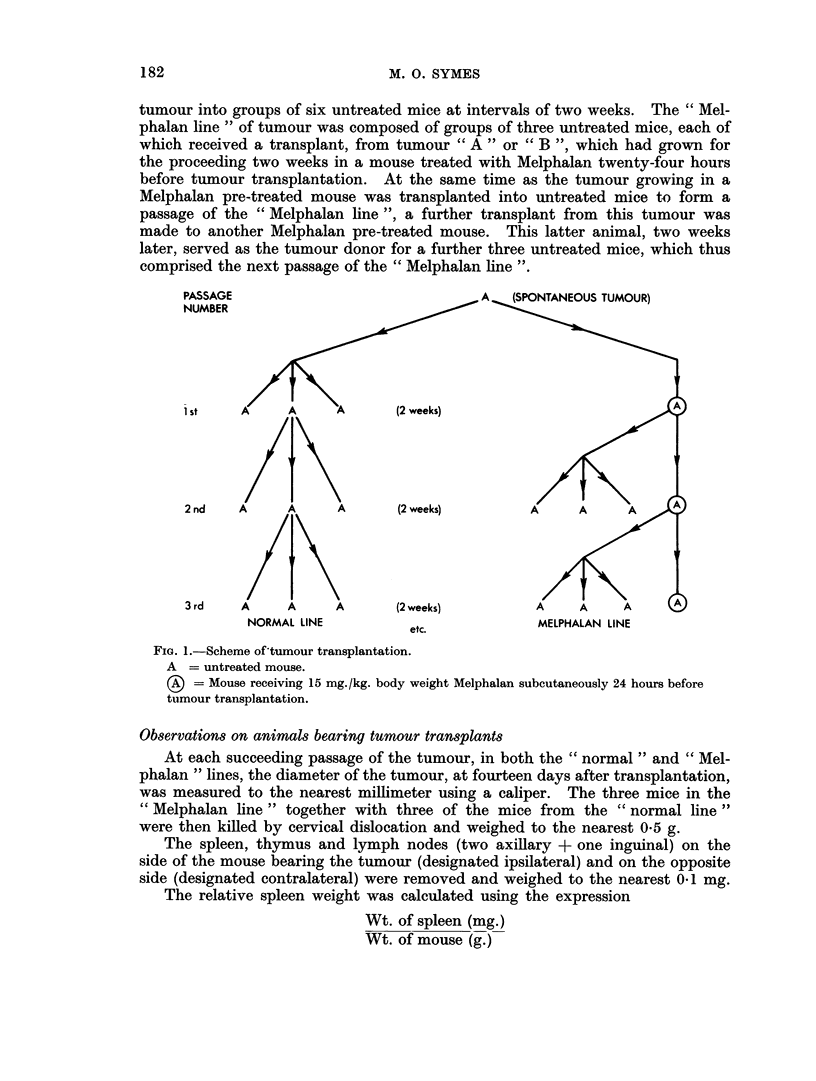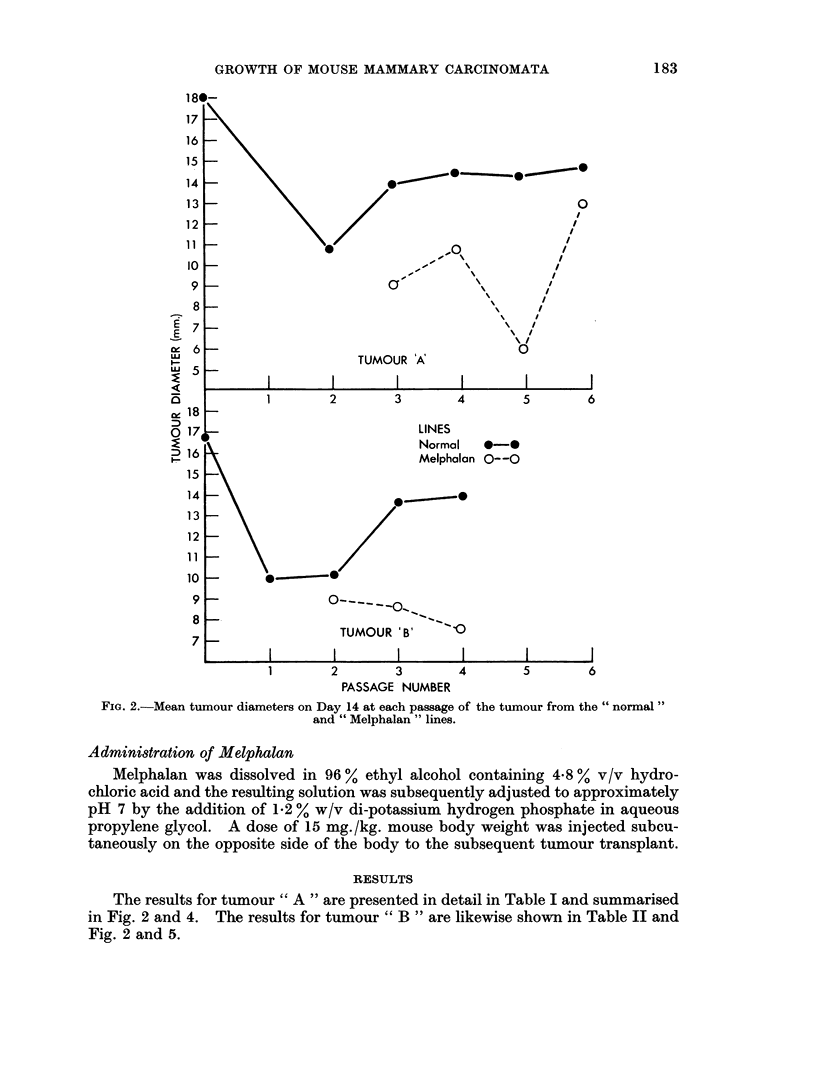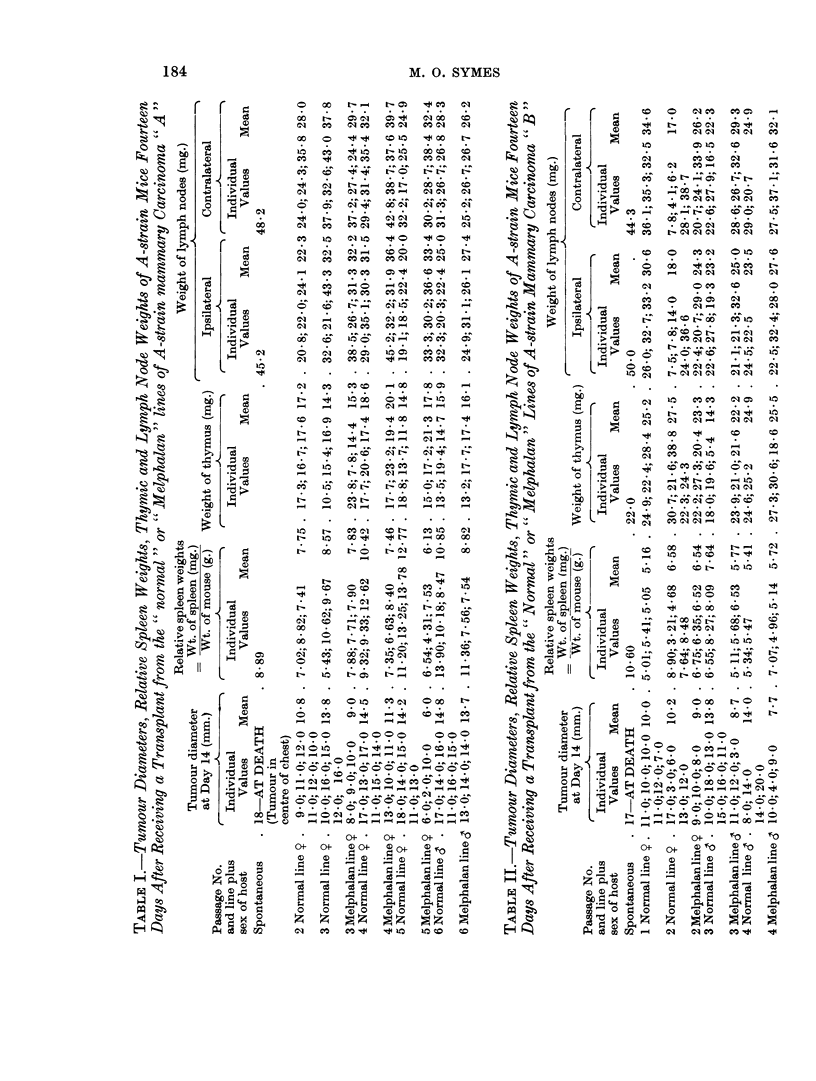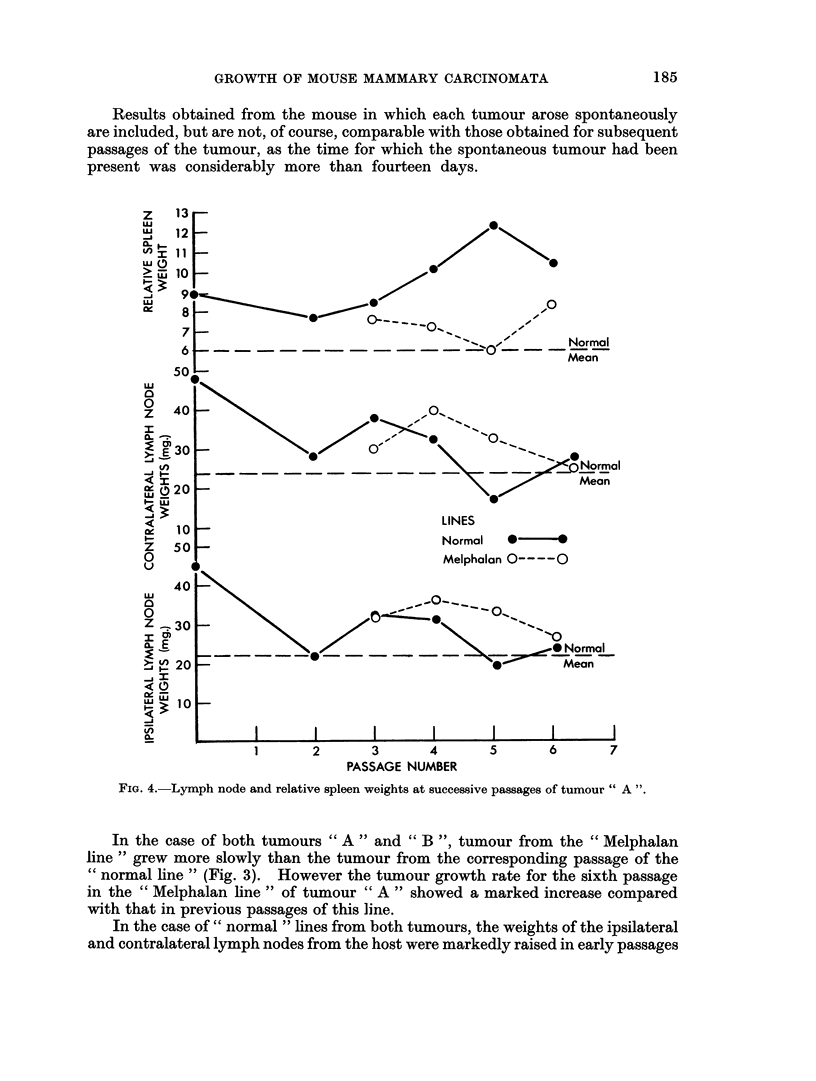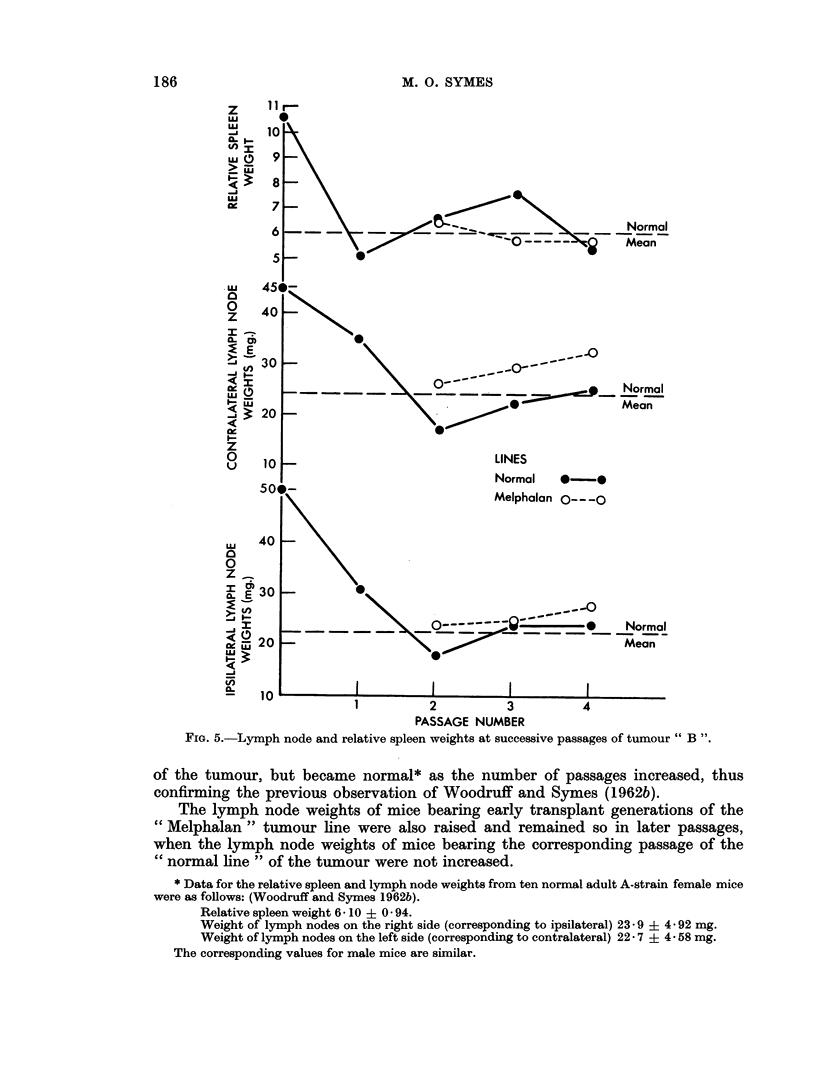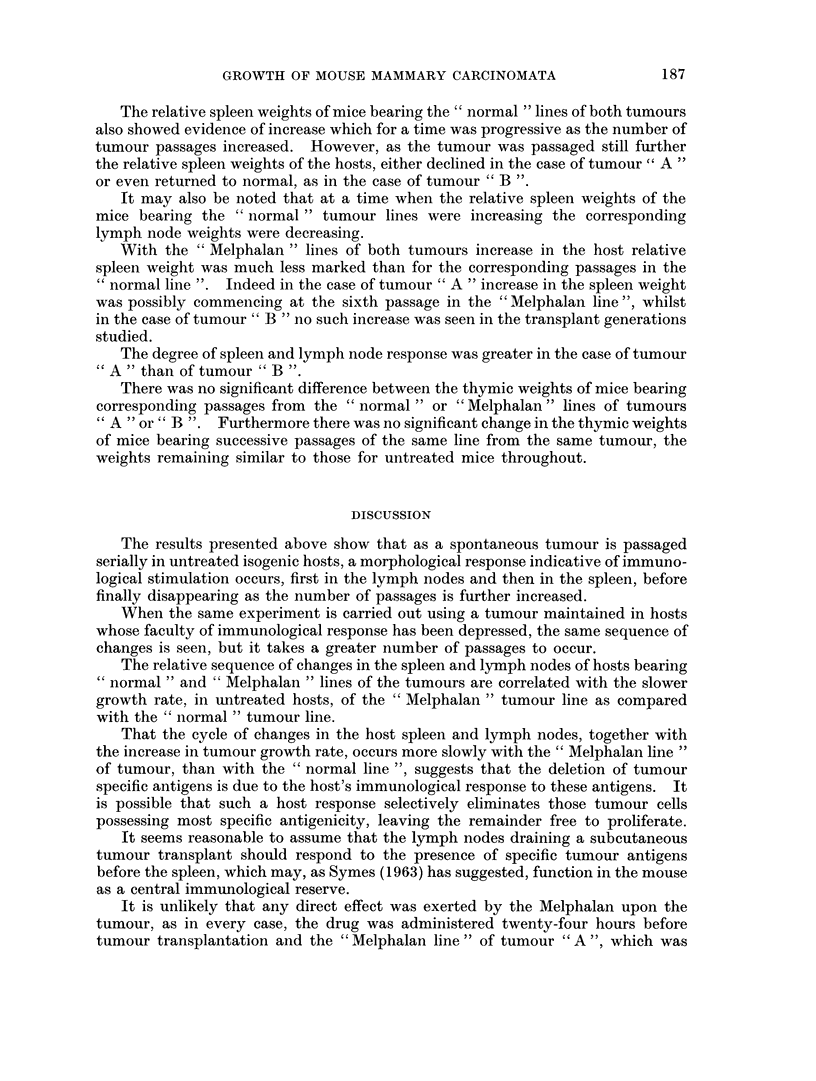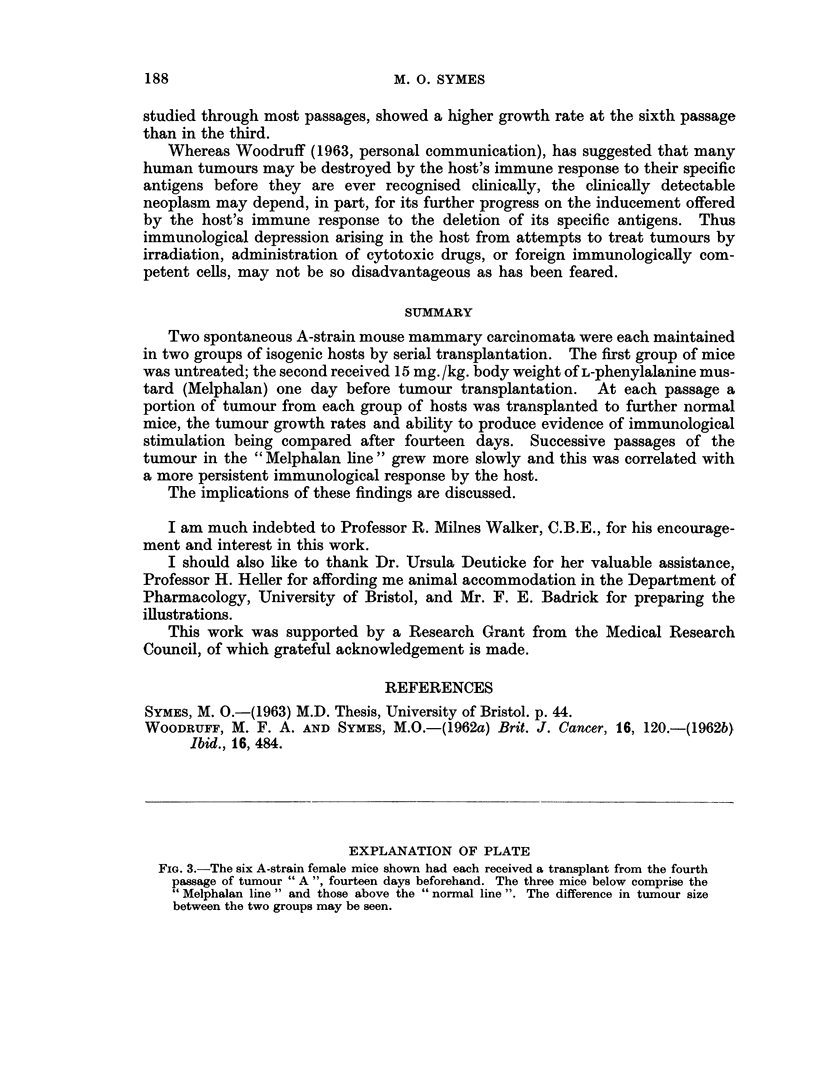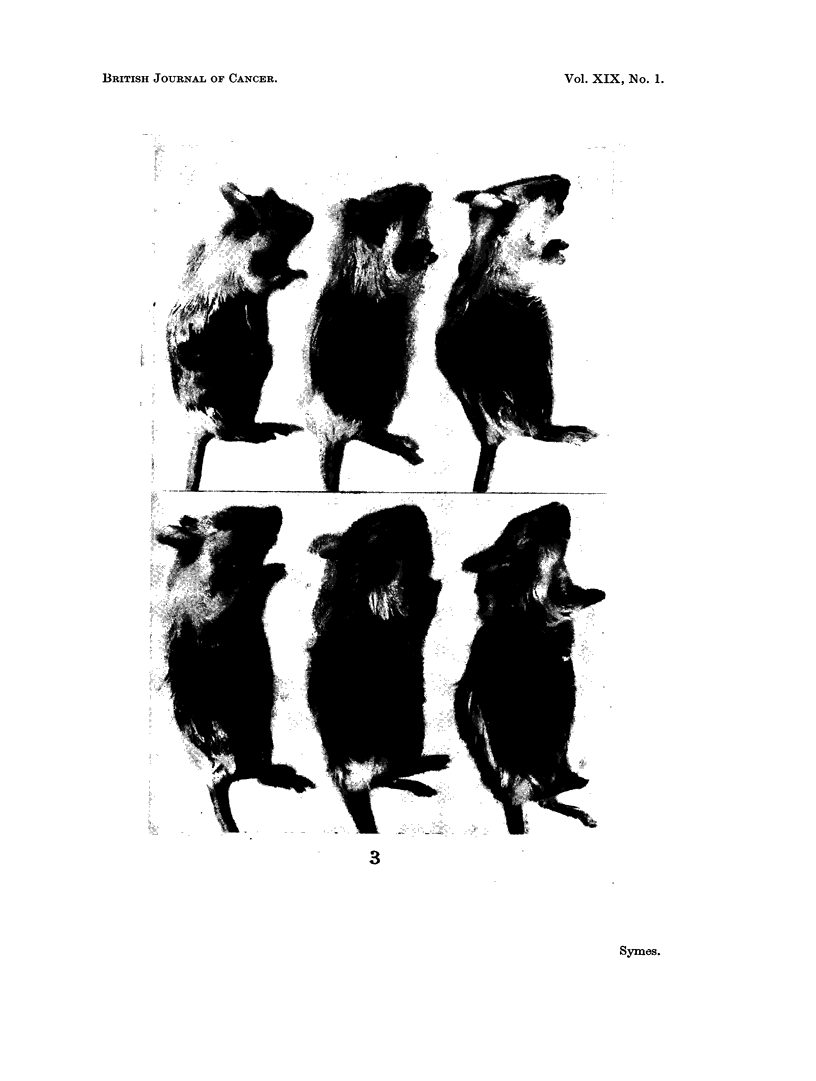# Further Observations on the Growth of Mouse Mammary Carcinomata in the Strain of Origin

**Published:** 1965-03

**Authors:** M. O. Symes

## Abstract

**Images:**


### FURTHER OBSERVATIONS ON THE GROWTH OF MOUSE MAMMARY CARCINOMATA IN THE STRAIN OF ORIGIN

### The Influence of the Host's Immunological Resistance on the Deletion of Tumour Specific Antigens

M. O. SYMES

*From the Department of Surgery, University of Bristol*

Received for publication October 3, 1964

It was reported by Woodruff and Symes (1962*a*, *b*) that the growth of spontaneous A-strain mouse mammary carcinomata, or early transplant generations thereof to isogenic hosts, was associated with hyperplasia of the spleen and lymph nodes, which, microscopically, was characteristic of immunological stimulation. It was suggested that this stimulation occurred in response to specific antigens possessed by the tumour. The finding that, following repeated transplantation within the strain of origin, the tumour no longer produced evidence of immunological stimulation in the host, led to the hypothesis that one of the characteristics of progressive malignant growth was the deletion of tumour specific antigens.

The purpose of the present communications is, first, to study the mechanism by which antigen deletion may occur, and second to correlate its occurrence with other evidence of increasing malignancy on the part of the tumour.

Theoretically deletion of tumour specific antigens could occur as a fundamental property of the malignant cells themselves, or as part of an adaptive response by the cells to immunological attack mounted against them by the host. An attempt has been made to distinguish between these possibilities by comparing the rates of antigen deletion shown by the same tumour maintained separately as two lines ; one in untreated isogenic hosts and the other in similar hosts whose immunological function had been depressed by prior administration of L-phenylalanine mustard (Melphalan).

### MATERIALS AND METHODS

*Mice and tumours studied*

Highly inbred A-strain mice of both sexes maintained in this department by strict brother × sister mating have been used throughout the investigation.

Two mammary carcinomata arising spontaneously in female A-strain mice were studied. The first tumour " A " (kindly supplied by Professor M. F. A. Woodruff) was obtained as a first generation transplant in an isogenic host, the second tumour " B " arose spontaneously in a member of the A-strain colony of this department.

*Mode of tumour transplantation*

The method of tumour transplantation was that described by Woodruff and Symes (1962*a*) and the scheme employed is shown in Fig. 1. The " normal line " of tumour was maintained by serial transplantation of the spontaneously occurring tumour into groups of six untreated mice at intervals of two weeks. The " Melphalan line " of tumour was composed of groups of three untreated mice, each of which received a transplant, from tumour " A " or " B ", which had grown for the proceeding two weeks in a mouse treated with Melphalan twenty-four hours before tumour transplantation. At the same time as the tumour growing in a Melphalan pre-treated mouse was transplanted into untreated mice to form a passage of the " Melphalan line ", a further transplant from this tumour was made to another Melphalan pre-treated mouse. This latter animal, two weeks later, served as the tumour donor for a further three untreated mice, which thus comprised the next passage of the " Melphalan line ".

FIG. 1.—Scheme of tumour transplantation.
A = untreated mouse.
Ⓐ = Mouse receiving 15 mg./kg. body weight Melphalan subcutaneously 24 hours before tumour transplantation.

*Observations on animals bearing tumour transplants*

At each succeeding passage of the tumour, in both the " normal " and " Melphalan " lines, the diameter of the tumour, at fourteen days after transplantation, was measured to the nearest millimeter using a caliper. The three mice in the " Melphalan line " together with three of the mice from the " normal line " were then killed by cervical dislocation and weighed to the nearest 0·5 g.

The spleen, thymus and lymph nodes (two axillary + one inguinal) on the side of the mouse bearing the tumour (designated ipsilateral) and on the opposite side (designated contralateral) were removed and weighed to the nearest 0·1 mg.

The relative spleen weight was calculated using the expression

$$\frac{\text{Wt. of spleen (mg.)}}{\text{Wt. of mouse (g.)}}$$

FIG. 2.—Mean tumour diameters on Day 14 at each passage of the tumour from the " normal " and " Melphalan " lines.

*Administration of Melphalan*

Melphalan was dissolved in 96% ethyl alcohol containing 4·8% v/v hydrochloric acid and the resulting solution was subsequently adjusted to approximately pH 7 by the addition of 1·2% w/v di-potassium hydrogen phosphate in aqueous propylene glycol. A dose of 15 mg./kg. mouse body weight was injected subcutaneously on the opposite side of the body to the subsequent tumour transplant.

### RESULTS

The results for tumour " A " are presented in detail in Table I and summarised in Fig. 2 and 4. The results for tumour " B " are likewise shown in Table II and Fig. 2 and 5.

Results obtained from the mouse in which each tumour arose spontaneously are included, but are not, of course, comparable with those obtained for subsequent passages of the tumour, as the time for which the spontaneous tumour had been present was considerably more than fourteen days.

FIG. 4.—Lymph node and relative spleen weights at successive passages of tumour " A ".

In the case of both tumours " A " and " B ", tumour from the " Melphalan line " grew more slowly than the tumour from the corresponding passage of the " normal line " (Fig. 3). However the tumour growth rate for the sixth passage in the " Melphalan line " of tumour " A " showed a marked increase compared with that in previous passages of this line.

In the case of " normal " lines from both tumours, the weights of the ipsilateral and contralateral lymph nodes from the host were markedly raised in early passages

FIG. 5.—Lymph node and relative spleen weights at successive passages of tumour " B ".

of the tumour, but became normal* as the number of passages increased, thus confirming the previous observation of Woodruff and Symes (1962*b*).

The lymph node weights of mice bearing early transplant generations of the " Melphalan " tumour line were also raised and remained so in later passages, when the lymph node weights of mice bearing the corresponding passage of the " normal line " of the tumour were not increased.

* Data for the relative spleen and lymph node weights from ten normal adult A-strain female mice were as follows: (Woodruff and Symes 1962*b*).

Relative spleen weight $6 \cdot 10 \pm 0 \cdot 94$.

Weight of lymph nodes on the right side (corresponding to ipsilateral) $23 \cdot 9 \pm 4 \cdot 92$ mg.

Weight of lymph nodes on the left side (corresponding to contralateral) $22 \cdot 7 \pm 4 \cdot 58$ mg.

The corresponding values for male mice are similar.

The relative spleen weights of mice bearing the " normal " lines of both tumours also showed evidence of increase which for a time was progressive as the number of tumour passages increased. However, as the tumour was passaged still further the relative spleen weights of the hosts, either declined in the case of tumour " A " or even returned to normal, as in the case of tumour " B ".

It may also be noted that at a time when the relative spleen weights of the mice bearing the " normal " tumour lines were increasing the corresponding lymph node weights were decreasing.

With the " Melphalan " lines of both tumours increase in the host relative spleen weight was much less marked than for the corresponding passages in the " normal line ". Indeed in the case of tumour " A " increase in the spleen weight was possibly commencing at the sixth passage in the "Melphalan line", whilst in the case of tumour " B " no such increase was seen in the transplant generations studied.

The degree of spleen and lymph node response was greater in the case of tumour " A " than of tumour " B ".

There was no significant difference between the thymic weights of mice bearing corresponding passages from the " normal " or "Melphalan" lines of tumours " A " or " B ". Furthermore there was no significant change in the thymic weights of mice bearing successive passages of the same line from the same tumour, the weights remaining similar to those for untreated mice throughout.

### DISCUSSION

The results presented above show that as a spontaneous tumour is passaged serially in untreated isogenic hosts, a morphological response indicative of immunological stimulation occurs, first in the lymph nodes and then in the spleen, before finally disappearing as the number of passages is further increased.

When the same experiment is carried out using a tumour maintained in hosts whose faculty of immunological response has been depressed, the same sequence of changes is seen, but it takes a greater number of passages to occur.

The relative sequence of changes in the spleen and lymph nodes of hosts bearing " normal " and " Melphalan " lines of the tumours are correlated with the slower growth rate, in untreated hosts, of the " Melphalan " tumour line as compared with the " normal " tumour line.

That the cycle of changes in the host spleen and lymph nodes, together with the increase in tumour growth rate, occurs more slowly with the " Melphalan line " of tumour, than with the " normal line ", suggests that the deletion of tumour specific antigens is due to the host's immunological response to these antigens. It is possible that such a host response selectively eliminates those tumour cells possessing most specific antigenicity, leaving the remainder free to proliferate.

It seems reasonable to assume that the lymph nodes draining a subcutaneous tumour transplant should respond to the presence of specific tumour antigens before the spleen, which may, as Symes (1963) has suggested, function in the mouse as a central immunological reserve.

It is unlikely that any direct effect was exerted by the Melphalan upon the tumour, as in every case, the drug was administered twenty-four hours before tumour transplantation and the "Melphalan line" of tumour "A", which was studied through most passages, showed a higher growth rate at the sixth passage than in the third.

Whereas Woodruff (1963, personal communication), has suggested that many human tumours may be destroyed by the host's immune response to their specific antigens before they are ever recognised clinically, the clinically detectable neoplasm may depend, in part, for its further progress on the inducement offered by the host's immune response to the deletion of its specific antigens. Thus immunological depression arising in the host from attempts to treat tumours by irradiation, administration of cytotoxic drugs, or foreign immunologically competent cells, may not be so disadvantageous as has been feared.

SUMMARY

Two spontaneous A-strain mouse mammary carcinomata were each maintained in two groups of isogenic hosts by serial transplantation. The first group of mice was untreated; the second received 15 mg./kg. body weight of L-phenylalanine mustard (Melphalan) one day before tumour transplantation. At each passage a portion of tumour from each group of hosts was transplanted to further normal mice, the tumour growth rates and ability to produce evidence of immunological stimulation being compared after fourteen days. Successive passages of the tumour in the "Melphalan line" grew more slowly and this was correlated with a more persistent immunological response by the host.

The implications of these findings are discussed.

I am much indebted to Professor R. Milnes Walker, C.B.E., for his encouragement and interest in this work.

I should also like to thank Dr. Ursula Deuticke for her valuable assistance, Professor H. Heller for affording me animal accommodation in the Department of Pharmacology, University of Bristol, and Mr. F. E. Badrick for preparing the illustrations.

This work was supported by a Research Grant from the Medical Research Council, of which grateful acknowledgement is made.

### REFERENCES

SYMES, M. O.—(1963) M.D. Thesis, University of Bristol. p. 44.

WOODRUFF, M. F. A. AND SYMES, M.O.—(1962*a*) *Brit. J. Cancer*, **16**, 120.—(1962*b*) *Ibid.*, **16**, 484.

---

EXPLANATION OF PLATE

FIG. 3.—The six A-strain female mice shown had each received a transplant from the fourth passage of tumour " A ", fourteen days beforehand. The three mice below comprise the " Melphalan line " and those above the " normal line ". The difference in tumour size between the two groups may be seen.

3

Symes.